# The Molecular Basis for Recognition of CD1d/α-Galactosylceramide by a Human Non-Vα24 T Cell Receptor

**DOI:** 10.1371/journal.pbio.1001412

**Published:** 2012-10-23

**Authors:** Jacinto López-Sagaseta, Jennifer E. Kung, Paul B. Savage, Jenny Gumperz, Erin J. Adams

**Affiliations:** 1 Department of Biochemistry and Molecular Biology, University of Chicago, Chicago, Illinois, United States of America; 2 Department of Chemistry, Brigham Young University, Provo, Utah, United States of America; 3 Department of Medical Microbiology and Immunology, University of Wisconsin School of Medicine and Public Health, Madison, Wisconsin, United States of America; 4 Committee on Immunology, University of Chicago, Chicago, Illinois, United States of America; National Jewish Medical and Research Center/Howard Hughes Medical Institute, United States of America

## Abstract

CD1d-mediated presentation of glycolipid antigens to T cells is capable of initiating powerful immune responses that can have a beneficial impact on many diseases. Molecular analyses have recently detailed the lipid antigen recognition strategies utilized by the invariant Vα24-Jα18 TCR rearrangements of iNKT cells, which comprise a subset of the human CD1d-restricted T cell population. In contrast, little is known about how lipid antigens are recognized by functionally distinct CD1d-restricted T cells bearing different TCRα chain rearrangements. Here we present crystallographic and biophysical analyses of α-galactosylceramide (α-GalCer) recognition by a human CD1d-restricted TCR that utilizes a Vα3.1-Jα18 rearrangement and displays a more restricted specificity for α-linked glycolipids than that of iNKT TCRs. Despite having sequence divergence in the CDR1α and CDR2α loops, this TCR employs a convergent recognition strategy to engage CD1d/αGalCer, with a binding affinity (∼2 µM) almost identical to that of an iNKT TCR used in this study. The CDR3α loop, similar in sequence to iNKT-TCRs, engages CD1d/αGalCer in a similar position as that seen with iNKT-TCRs, however fewer actual contacts are made. Instead, the CDR1α loop contributes important contacts to CD1d/αGalCer, with an emphasis on the 4′OH of the galactose headgroup. This is consistent with the inability of Vα24− T cells to respond to α-glucosylceramide, which differs from αGalCer in the position of the 4′OH. These data illustrate how fine specificity for a lipid containing α-linked galactose is achieved by a TCR structurally distinct from that of iNKT cells.

## Abstract 2

Human Vα24− CD1d-restricted T cells use variation in their CDR1α loop to respond to lipid antigens presented by CD1d, altering their specificities from that of invariant natural killer T cells.

## Author Summary

Certain lineages of T cells can recognize lipids as stimulatory antigens when presented in the context of CD1 molecules. We know how most Natural Killer T (NKT) cells react with this unusual ligand because they use a single invariant T cell receptor (TCR) alpha chain to do the job. NKT cells place particular emphasis on their CDR3α and CDR2β loops in recognition of antigen—these complementarity determining regions (CDRs) are the hypervariable parts of the TCR that *“complement”* an antigen's shape. How do these other T cells recognize closely related yet distinct lipid antigens? Here we show that human CD1d-restricted T cells, typically called Vα24− T cells due to their use of diverse Vα domains in their TCRs, use similar molecular strategies to respond to lipid antigens presented by CD1d. To this end we present a 2.5 Å complex structure of a Vα24− TCR complexed with CD1d presenting the protypical lipid, α-galactosylceramide (αGalCer). The TCR examined in this study notably shifts its binding slightly, placing more emphasis on the interaction with the CDR1α loop as revealed through alanine scanning mutagenesis. This shift explains the inability of these T cells to respond to lipids that vary at this site of contact (the 4'OH), like the related α-linked glucosylceramide. These results provide a molecular basis for the fine-specificity of different CD1d-restricted T cell lineages.

## Introduction

Natural killer T (NKT) cells are a highly conserved lineage of T lymphocytes found in both human and mice that are involved in the modulation of the immune response in autoimmunity, infection, and tumor development [1]. Unlike conventional CD4^+^ and CD8^+^ αβ T cells that recognize peptides presented by MHC molecules, NKT cells are reactive to a broad range of self and foreign lipids displayed by the MHC class I–like molecule CD1d [2],[3]. This reactivity is initiated by the recognition of the CD1d-lipid complex via the NKT T cell receptor (NKT-TCR) followed by Th1 and/or Th2 biased cytokine secretion that can regulate the activity of other immune cells such as conventional αβ T cells, B cells, and Natural Killer (NK) cells [4].

The most extensively studied NKT cells in humans and mice are invariant (iNKT) or type I NKT cells that express TCRs composed of a highly conserved α chain encoded by a Vα24-Jα18 rearranged gene segment in humans and Vα14-Jα18 in mice. This invariant α chain is covalently paired with a β chain in which the variable region is encoded in humans by the Vβ11 gene and can be Vβ8, Vβ7, or Vβ2 in mice [1]. NKT cells expressing these TCRs have a pre-activated phenotype that is due to the expression of the transcription factor pro-myelocytic leukemia zinc finger (PLZF) [5],[6] and are also characterized by high reactivity towards the potent stimulatory lipid antigen α-galactosylceramide (αGalCer) [7]. In both humans and mice there are additional classes of T cells that respond to CD1d, one that expresses diverse TCRs but do not respond to αGalCer; these are generally called Type II or non-invariant NKT cells [8]. These NKT cells are typically reactive to lipid antigens such as sulfatide and use an entirely different molecular strategy for recognizing the CD1d/lipid complex [9],[10]. A third group of T cells exist that do respond to CD1d presenting αGalCer and also express TCRs different from that of the iNKT-TCR. In mice these NKT cells express a TCR comprised of a Vα10-Jα50/Vβ8 pair [11]. These cells are called Vα10 NKT cells and show a preference for α-glucosylceramide (αGlcCer) over αGalCer; indeed, Vα10 NKT cells can produce a several magnitudes greater cytokine response relative to iNKT cells when stimulated by the related α-glucuronosyldiacylglycerl (α-GlcA-DAG) [11].

In humans this third group of CD1d reactive T cells express TCRs with many different Vα domains joined with Jα18, paired with the Vβ11 domain [12],[13]. In contrast to both Type I and Type II NKT cells, these T cells do not typically express CD161, a Natural Killer cell marker found on NKT cells [13]. They have been called Vα24− NKT cells or CD1d-restricted, Vα24− T cells due to their use of alternative Vα domains rearranged to Jα18, paired with the Vβ11 domain in their TCRs. These cells are found in all individuals sampled [13] at appreciable frequency (∼10^−5^) [14] and express either the CD8αβ or CD4 co-receptors, can be cytotoxic, and can secrete IL-2, IFN-γ, and IL-13 (and in some cases IL-4) [13]. In contrast to human iNKT cells, they express low to intermediate levels of PLZF and have a naïve phenotype [14]. Importantly, these NKT cells have shifted lipid specificities from that of iNKT cells with an inability to recognize and respond to αGlcCer [12]. The distinctive difference in reactivity between αGalCer and αGlcCer suggests that this population of NKT cells focuses on a different repertoire of lipid antigens than those of iNKT cells.

Despite the variability that exists in NKT cell populations, most of our current knowledge of NKT cell recognition of antigen derives from structural studies that have focused on self and foreign lipid antigen recognition by Type I iNKT TCRs [15]. iNKT-TCRs recognize, through their complementary determining regions (CDR) loops, a composite surface composed of the α-helices of CD1d and the solvent exposed head group of the CD1d-presented lipid antigens. The CDR3α loop plays a prominent, conserved role in CD1d-lipid recognition, predominantly via residues encoded by the Jα18 segment, which is found in all iNKT TCRs. There are also important contributions from the CDR1α and CDR2β loops, which explain the restricted use of specific Vα and Vβ domains (which encode the CDR1 and CDR2 loops) [16],[17]. For each Vβ chain used in mouse, the docking of iNKT-TCRs on the CD1d/lipid antigen surface is remarkably conserved [18],[19], indeed variation of the lipid antigen is accommodated mainly through structural modifications of the lipid antigen as opposed to changes in the iNKT TCR footprint [20]–[24]. The number of human iNKT TCR complex structures are fewer yet reflect some flexibility in docking of the iNKT TCR depending on the lipid antigen [16],[19],[23],[25], yet appear to be similarly anchored via conserved positioning of the CDR3α loop.

The crystal structure of a murine Vα10 NKT TCR in complex with murine CD1d-αGlcCer [11] has shed light onto the molecular mechanisms that murine non-canonical NKT TCRs use to recognize CD1d. Despite significant sequence divergence in the α chain amino acid sequence (40% sequence identity), the Vα10 NKT TCR assumes a very similar docking mode to that of the iNKT TCR on CD1d. However, unlike the iNKT TCR, all CDR loops of the Vα10 NKT TCR contribute to CD1d/αGlcCer recognition, with seemingly important contacts being contributed by the CDR2β and CDR3β loops. Thus the two Vα chains of these divergent murine NKT cell populations (iNKT and Vα10) have convergently evolved a similar molecular strategy for recognizing CD1d. Recently, crystal structures of the Type II NKT TCR recognition of CD1d presenting sulfatide [9] and lysosulfatide [10] provided an interesting contrast to the conserved recognition of CD1d by the iNKT and murine Vα10 TCRs. The Type II TCRs use all six CDR loops in CD1d/ligand engagement and dock on a separate site on CD1d, concentrating on residues surrounding the A′ pocket. Thus, NKT cells have a range of docking modes used in CD1d/ligand engagement.

Structural data on NKT cell recognition in humans remains limited, and information of how Vα24− T cells recognize CD1d/lipid is, to our knowledge, absent. To better understand how this functionally distinct human T cell population recognizes CD1d/lipid, we have co-crystallized a Vα24− TCR with CD1d/αGalCer and present here the structure of this complex resolved to 2.5 Å resolution. This structure provides an excellent model by which to understand how functionally distinct human T cells, via their TCR, can recognize CD1d with a shifted specificity from that found in the iNKT cell population.

## Results

### Structure of a Vα24− TCR in Complex with CD1d/αGalCer

In order to understand the molecular basis of Vα24− TCR recognition of CD1d, we expressed a soluble, heterodimeric version of the extracellular domains of the J24.N22 TCR [12], which uses the Vα3.1 (TRAV17) gene segment rearranged with Jα18 complexed with Vβ11, in insect cells. The purified TCR was co-crystalized with recombinant, soluble CD1d loaded with αGalCer; X-ray data were collected to 2.5 Å, and the structure was solved via molecular replacement. Data collection and refinement statistics are listed in Table 1. One TCR/CD1d/αGalCer ternary complex was identified in the asymmetric unit. All components of this complex were well resolved in the electron-density, enabling unambiguous assignment of TCR-CD1d/lipid antigen contacts.

**Table 1:** Data collection and refinement statistics (molecular replacement).

Data Collection	Vα24− TCR-CD1d-αGalCer
Space group	P 1 2_1_ 1
Cell dimensions	
*a*, *b*, *c* (Å)	57.11, 72.57, 113.75
β (°)	103.3
Resolution (Å)	50–52.55 (2.59–2.55)
*R*_sym_	0.065 (0.495)
*I*/σ*I*	24.0 (2.4)
Completeness (%)	100 (100)
Redundancy	3.8 (3.7)
**Refinement**	
Resolution (Å)	2.55
Total number of reflections	109,937
Number of unique reflections	28,919
*R*_work_/*R*_free_	0.208/0.266
Number of atoms	
Protein	6,132
Ligand/ion	148
Water	126
*B*-factors	
Protein	60.7
Ligand/ion	71.9
Water	53.9
R.m.s. deviations	
Bond lengths (Å)	0.002
Bond angles (°)	0.570

Values in parentheses are for highest resolution shell.

### Sequence Variability between Vα24+ and Vα24− TCRs

Table 2 presents a comparison between the amino acid sequences of the α and β CDR loops of the Vα24− (Vα3.1+) TCR studied here and an iNKT Vα24+ TCR studied previously [25]. Vα3.1 and Vα24 share 46% amino acid identity overall, with only 33% (2/6) identity at the CDR1α and 15% (1/7) at the CDR2α loop. However, the shared usage between these TCRs of the Jα18 segment and the canonical DRGSTLGR motif that it encodes gives high sequence identity to the CDR3α loops of these TCRs with different residues encoded only at the Vα-Jα junction, with ATY and VVS motifs in the Vα24− and Vα24+ TCRs, respectively. The Vβ11 domain is also shared between these TCRs; therefore, the CDR1β and CDR2β sequences are identical. However, the rearranged CDR3β loops differ due to differences introduced during the rearrangement process.

**Table 2:** Alignment of the Vα24− and Vα24+ NKT TCR CDR loop sequences.

TCR	CDR1α	CDR2α	CDR3β
J24N.22 (Vα24−)	TSINN	IRSNERE	ATY DRGSTLGRLYFGRGTQLTVWP
iNKT Vα24+	VSPFSN	MTFSENT	VVS DRGSTLGRLYFGRGTQLTVWP

TCR	CDR1β	CDR2β	CDR3β
J24N.22 (Vβ11)	MGHDK	YSYGVNST	CASSE NSGTGRI YEQYFGPGTRLTVT
iNKT Vβ11	MGHDK	YSYGVNST	CASS GLRDRGL YEQYFGPGTRLTVT

### Recognition of CD1d/αGalCer by the Vα24− TCR

**Figure 1:**
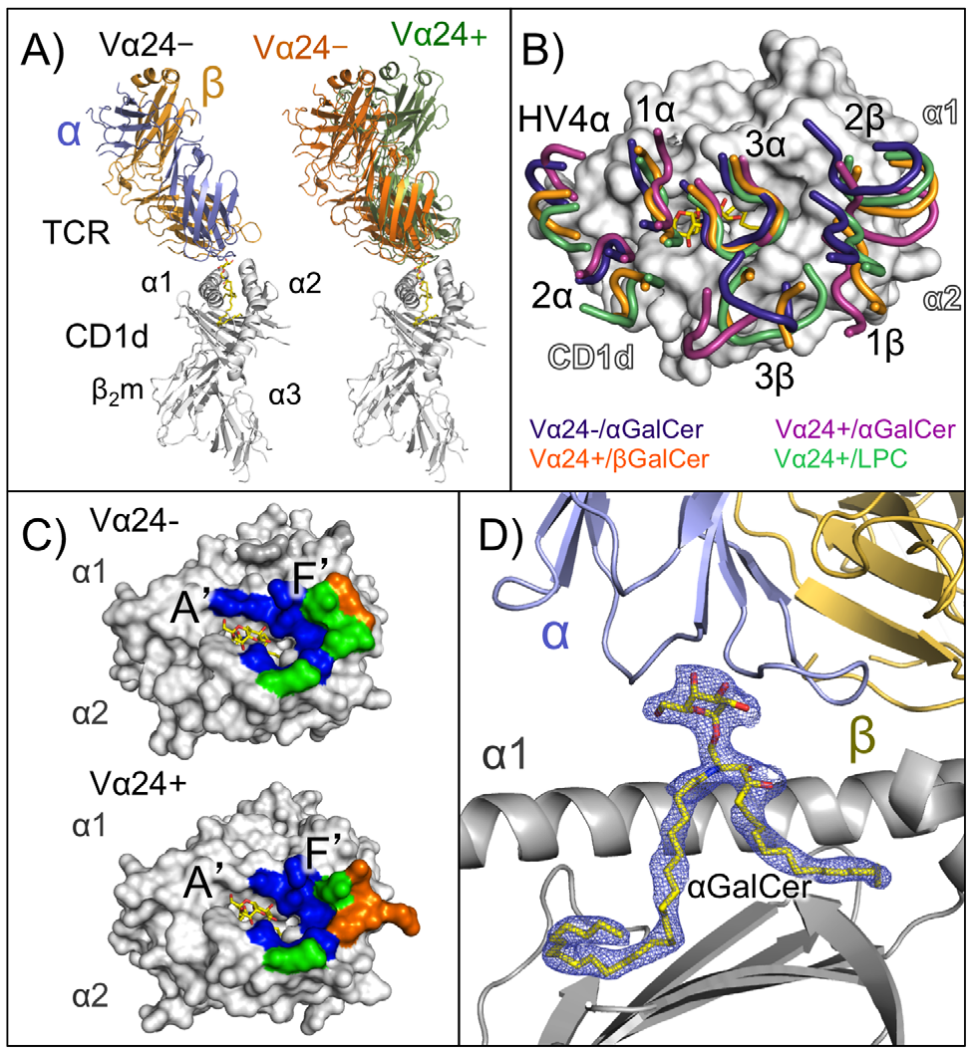
Complex structure of the Vα24− TCR with CD1d-αGalCer. (A) Left panel, ribbon representation of the human Vα24− TCR J24.N22 (slate, α chain; orange, β chain) in complex with human CD1d-β_2_m (ribbon, white) and αGalCer (sticks, yellow). Right panel, the Vα24− TCR-CD1d-αGalCer complex (orange) is shown superimposed with a Vα24+ NKT TCR-CD1d-αGalCer complex (PDB ID: 3HUJ; TCR in green) [19]. Complexes were aligned via the main-chain CA carbons of the CD1d heavy chain. (B) Positioning of the four different human NKT TCR loops on the CD1d-ligand surface: purple, Vα24− TCR; orange, Vα24+ NKT TCR-CD1d-βGalCer (PDB ID: 3SDX) [23]; berry, Vα24+ NKT TCR-CD1d-αGalCer complex; and green, the iNKT TCR-CD1d-LPC complex (PDB ID: 3TZV) [25]. Shown is CD1d (white)-αGalCer (yellow) from the Vα24− TCR-CD1-αGalCer complex. (C) Upper panel, footprint of the Vα24− TCR on the surface of CD1d-αGalCer. Residues that are contacted by the TCR α chain, β chain, or both are colored in blue, orange, and green, respectively. Lower panel, footprint of the Vα24+ NKT TCR on the surface of CD1d-αGalCer; colors of CD1d are as for the Vα24− TCR. (D) Electron density of the αGalCer ligand in the Vα24− TCR-CD1d-αGalCer complex. Electron density, shown as a blue mesh, corresponds to a composite omit map (2Fo–Fc) contoured at 1σ around the αGalCer ligand (yellow). CD1d is shown in grey ribbons, and the α2 helix has been omitted to facilitate the visualization of the ligand. The TCR α and β chains in light blue and yellow-orange, respectively.

Overall, the Vα24− TCR recognizes CD1d/αGalCer with the α and β chains oriented on CD1d in a parallel fashion unlike the typical diagonal mode of MHC-I peptide-TCR complexes and similar to that of iNKT-TCR and Vα10 NKT-TCR in complex with CD1d/αGalCer (Figure 1A and 1B) [11],[16],[19]. However, the binding angle of the Vα24− TCR in relation to the CD1d/αGalCer surface is more acute than the almost perpendicular orientation observed with the Vα24+ iNKT TCR-CD1d/αGalCer structure (Figure 1A) [16],[19]. The CDRα loops adopt a similar yet slightly shifted footprint for the α-chain, yet the β-chain CDR loop positioning is counter-clockwise rotated compared with the Vα24+ TCR complexed with αGalCer [16],[19], which is even more extreme than rotations observed in structures of human NKT-TCRs complexed with CD1d presenting LPC or βGalCer (Figure 1B) [23],[25]. The TCR-CD1d-lipid contacts mostly fall in the F′ pocket area of the CD1d molecule (Figure 1C), where there are slight differences in TCR contact surface between the Vα24− and Vα24+. The total buried surface area (BSA) between the Vα24− TCR and the CD1d-αGalCer complex was 747 A^2^, which is slightly smaller than the previously reported interface area for the Vα24+ TCR, ∼910 A^2^. This difference is more pronounced in the β-chain loops with ∼37% less contribution in the Vα24− complex (205.7 A^2^ versus 325.3 A^2^ for the Vα24− and Vα24+, respectively).

### αGalCer Positioning in the Complex with the Vα24− TCR

The conformation and positioning of αGalCer presented by CD1d is almost identical in both complexes with the Vα24+ and the Vα24− TCRs. The sphinosine base and acyl chain of αGalCer fall in the F′ and A′ pockets, respectively (Figure 1D). The αGalCer headgroup also adopts a very similar conformation, with solvent exposed with the sugar oxygens displayed for recognition by the TCR. The conformation of the α helical side chains of CD1d were also highly conserved between the Vα24+ and Vα24− complex structures, with only a few exceptions that are noted later in the text.

### Convergent Recognition Strategy of a Vα24− TCR

**Figure 2:**
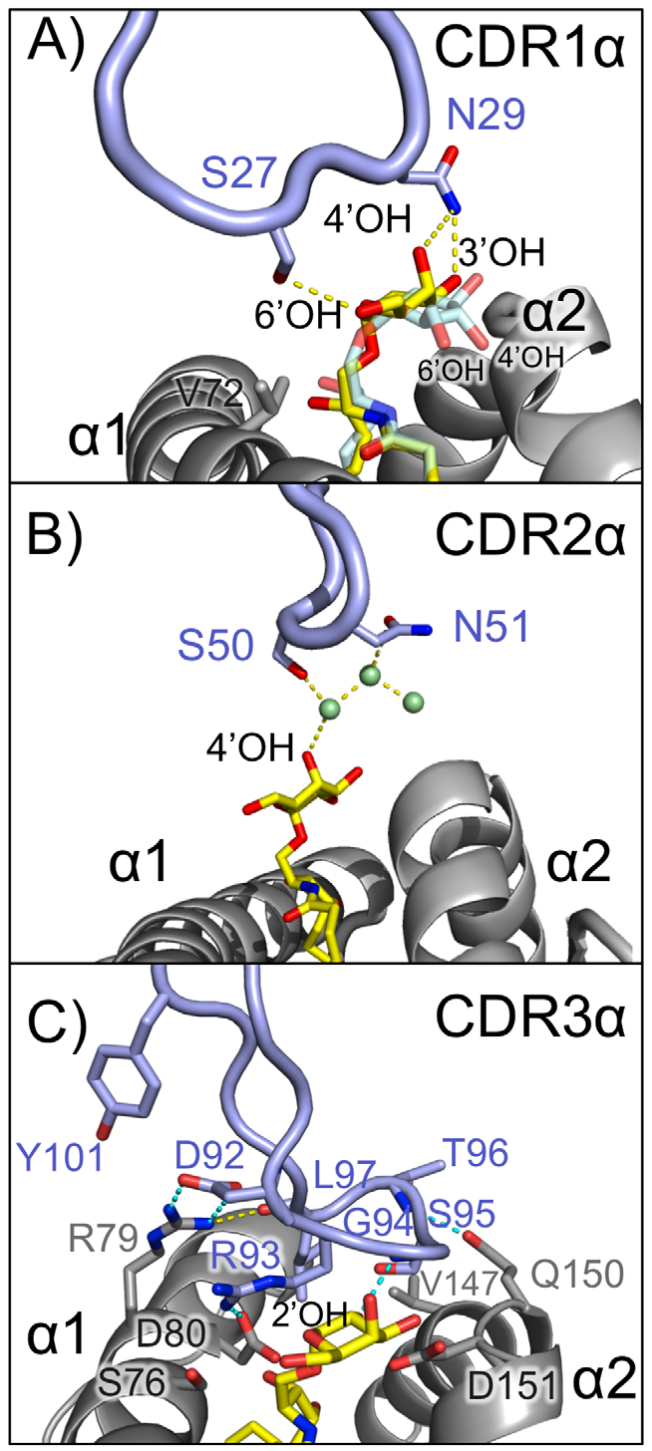
Unique and conserved contacts of the Vα24− TCR CDRα loops with CD1d-αGalcer. Contacts made by the CDR1α, CDR2α, and CDR3α loops to CD1d− αGalCer are represented in (A), (B), and (C) respectively. CD1d is shown as grey ribbons, TCR CDRα loops in light blue, and αGalCer is represented as yellow sticks. A model of αGlcCer generated via superposition of the CD1d/αGlcCer structure (PDB ID: 3ARG) [35] is shown in cyan in (A) in comparison with αGalCer. Positions of the 4′OH and 6′OH of αGlcCer are indicated. Water molecules in (B) are displayed as pale-green spheres. Hydrogen bonds (≤3.3 Å) are shown as yellow dashed lines. (C) Conserved hydrogen bonds between the Vα24− and Vα24+ NKT TCR CDR3α loops are shown as dashed lines colored cyan.

In all three human iNKT TCR-CD1d/lipid complexes that have been resolved to date, the CDR1α loop makes important contacts with the lipid headgroup [16],[19],[23],[25]. In recognition of αGalCer and βGalCer the O^γ^ of Ser30 and the mainchain carbonyl oxygen of Phe29 make hydrogen-bonds (some water-mediated) with the 3′OH of αGalCer and βGalCer, and in the case of LPC, the O^γ^ Ser27 and the mainchain carbonyl oxygen of Phe29 establish hydrogen bonds with the phosphate oxygens of the phosphorylcholine headgroup. Pro28 establishes van der Waals (VDW) contacts with the galactose headgroup; mutagenesis of this residue has a marked effect on recognition but is likely due to global structural changes in the conformation of the TCR as this mutation also disrupted binding of a conformational-specific antibody [17]. In our structure the Vα24− CDR1α loop is slightly shifted from the Vα24+ CDR1α loop (Figure 1B); therefore, the equivalent structural positions to the Vα24+ S_27_P_28_F_29_S_30_ motif are T_26_S_27_I_28_N_29_ in Vα24−. Despite the chemical and structural differences of the CDR1α loops between these TCRs, specific side-chain-mediated hydrogen bonds are still formed in the Vα24− CDR1α loop, both with the galactose headgroup of αGalCer and through VDW contacts with CD1d's Val72 (Figure 2A and Table 3). The shifted position of Ser27 in this complex enables a hydrogen bond between its O^γ^ with the 6′OH of αGalCer, whereas the N^δ2^ of Asn29 hydrogen bonds with the 3′OH and 4′OH of αGalCer and Asn29 also forms VDW contacts with the galactose headgroup. Therefore, alternative residues in the CDR1α loop are effectively used in recognition of αGalCer with a focus on the 4′OH of the galactose ring, with a novel contact with CD1d also noted.

**Table 3:** Atomic contact comparison of iNKT-TCRs, CD1d, and lipid ligands.

	Contacts Between CDR1α and αGalCer	
CDR1α	αGalCer	Bond Type
Ser27	O5A	VDW
Ser27O^γ^	O5A	HB
Asn29N^δ2^	O3A, O4A	HB
Asn29	O4A, C3A	VDW

	Contacts Between CDR3α and αGalCer	
CDR3α	αGalCer	Bond Type
Asp92	C1A	VDW
Arg93	C2A, O2A, O3	VDW
Gly94	C2A	VDW
Gly94	O2A, O3A, C2A	VDW

	Contacts Between CDR1α and CD1d	
CDR1α	CD1d	Bond Type
Ser27	Val72	VDW

	Contacts Between CDR2α and αGalCer	
CDR2α	CD1d	Bond Type
Ser50O	O4A	Water-mediated HB
Asn51O^δ1^	O4A	Water-mediated HB

	Contacts Between CDR3β and CD1d	
CDR3β	CD1d	Bond type
Ser97O	Gln150N^ε2^	HB (3.67)
Ser97	Gln150	VDW

	Contacts Between CDR2β and CD1d	
CDR2β	CD1d	Bond Type
Tyr48	Glu83, Lys86	VDW
Tyr48O^η^	Glu83O^ε1^, Glu83O^ε2^, Lys86N^ζ^	HB
Tyr50	Glu83, Met87	VDW
Tyr50O^η^	Glu83 O^ε1^	HB
Glu56	Lys86	VDW
Glu56	Lys86	VDW

	Contacts Between CDR3α and CD1d	
CDR3α	CD1d	Bond Type
Asp92	Arg79	VDW
Asp92 O^δ1^	Arg79 N^η1^, Arg79 N^η2^	SB
Asp92 O^δ2^	Arg79 N^η1^, Arg79 N^η2^	SB
Arg93	Ser76, Arg79, Asp80	VDW
Arg93N^η1^	Asp80 O^δ1^, Asp80 O^δ2^	SB
Gly94	Gln150, Asp151	VDW
Ser95	Val147, Gln150	VDW
Thr96^N^	Gln150 O^ε1^	HB
Thr96	Val147, Gln150	VDW
Leu97O	Arg79 N^η1^	HB
Leu97	Asp80, Glu83, Phe84, Met87, Val147	VDW
Arg99	Arg79	VDW
Tyr101	Arg79	VDW

We have also noted residues in the CDR2α loop that make water-mediated contacts with the αGalCer galactose headgroup: Ser50 and Asn51 both establish water-mediated hydrogen bonds with the 4′OH of αGalCer (Figure 2B). In the other human complexes, Phe51 of the Vα24+ CDR2α loop makes VDW contacts with both βGalCer and LPC, however hydrogen bonds have not been noted for the CDR2α loop of Vα24+ TCRs. In contrast to the sequence and contact differences at the CDR1α and CDR2α loops, the residues of the CDR3α loop in the Vα24− TCRs adopt a similar conformation to that of the Vα24+ iNKT TCRs (Figure 2C). Yet despite the similarity in footprint, the Vα24− CDR3α loop establishes fewer contacts with CD1d and αGalCer than does the CDR3α loop of the iNKT TCR (Table 3) (25 instead of 32, respectively, for CD1d and eight instead of 19, respectively, for αGalCer). There are fewer hydrogen bonds (two versus eight with CD1d and one versus four with αGalCer) and, in the case of αGalCer, fewer than half (seven versus 15) VDW contacts of those observed in the Vα24+ complex. The residues of the Vα24+ CDR3α were previously shown to be energetically critical for CD1d/αGalCer recognition [17], a finding recapitulated in our data (discussed further below) despite the lower contact number.

### A Shifted Vα24− TCR β Chain Maintains Conserved Contacts through the CDR2β

**Figure 3:**
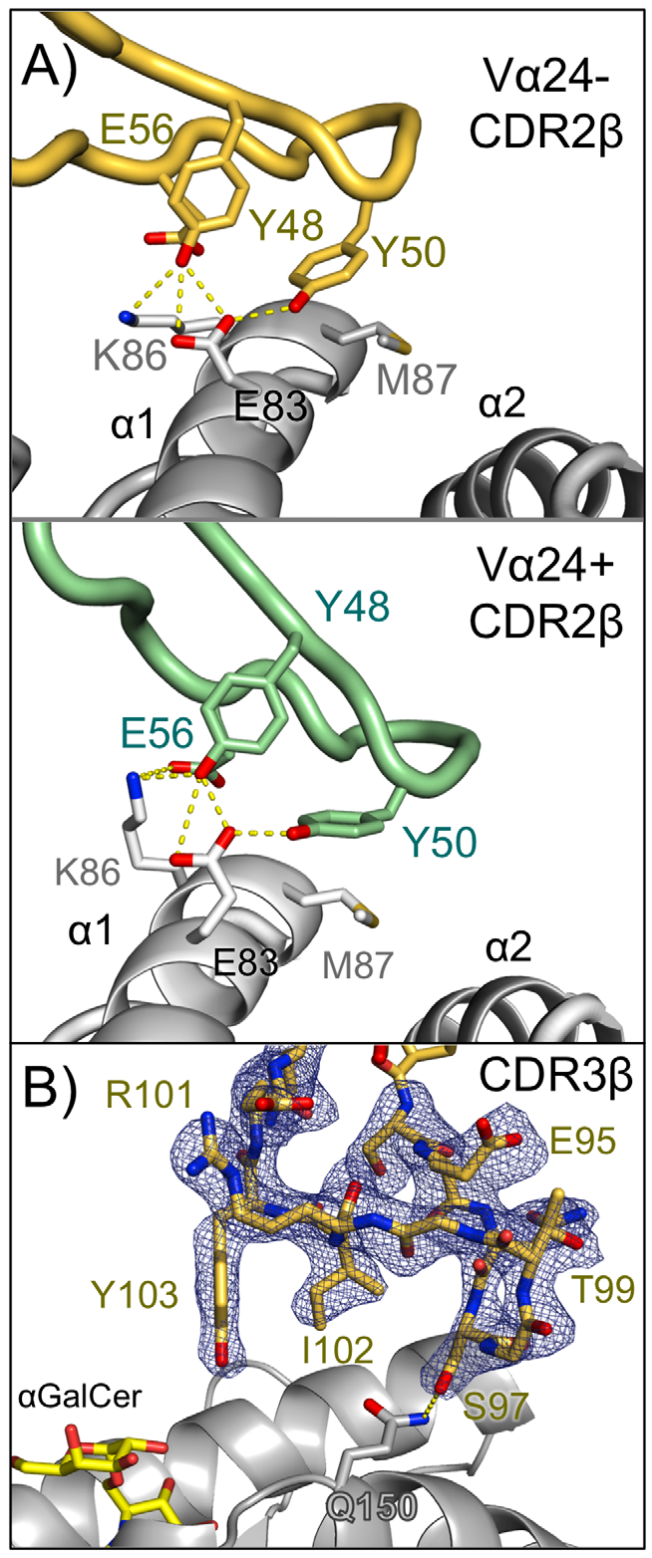
The role of the TCR β chain in Vα24− TCR engagement of CD1d-αGalCer. (A) Contacts made by the CDR2β loops of the Vα24− and Vα24+ NKT TCRs with CD1d. CD1d is shown as grey ribbons and the Vα24− and Vα24+ NKT TCR CDR2β loops in yellow-orange and pale-green color, respectively. Hydrogen bonds (≤3.3 Å) and salt bridges are shown as yellow dotted lines for the Vα24− and Vα24+ NKT TCRs. (B) Electron density (Fo-Fc omit map, contoured at 3σ) for the CDR3β loop of the Vα24− TCR is shown as blue mesh together with the CDR3β in stick representation in yellow-orange; CD1d is shown in grey ribbons and αGalCer in yellow sticks. Potential H-bond is displayed as dotted, yellow line.

While the CDR3α loop serves to anchor human iNKT TCRs on the CD1d/lipid platforms with highly similar conformations [16],[19],[23],[25], the remaining loops have demonstrated rotational flexibility in how they are positioned over the CD1d/lipid surface, in particular at the CDR2β, which establishes energetically critical contacts with CD1d [17]. A similar rotation is seen in the Vα24− TCR docking on the CD1d/αGalCer platform in the complex structure presented here (Figure 1B and Figure 3A). As in the Vα24+ complexes, the involvement of the CDR2β loop in CD1d binding is predominantly mediated by Tyr48 and Tyr50. Despite an average shift of 4.6 Å between the Vα24− and Vα24+ CDR2β CA backbones, the rotationally flexible tyrosine side chains maintain highly similar contacts between the two complexes (Figure 3A). Glu83 on CD1d takes a central role in contact with the CDR2β in both complexes, establishing a hydrogen-bonded network with both Tyr48 and Tyr50 hydroxyls. Met87 also contributes VDW contacts with Tyr50 in both complexes. However, in contrast to the Vα24+ complex, where Glu56 of the CDR2β establishes a robust salt-bridge with Lys86 of CD1d (3.7 Å distance), in the Vα24− complex Lys86 has shifted such that is it 4.6 Å from Glu56 (Figure 3A). Thus, the critical contacts of the CDR2β loop are maintained in the Vα24− complex despite large main chain shifts of the CDR2β backbone.

The highly variable CDR3β loop has been demonstrated to confer reactivity to specific lipids presented by CD1d by both human [26] and mouse [27] iNKT cells. In the Vα24− complex, the CDR3β loop is well resolved in the electron density and establishes only one weak hydrogen bond and a VDW contact with Gln150 on CD1d's α2-helix via Ser97 (Figure 3B). Thus, unlike the murine Vα10 NKT TCRs, which have CDR3β sequence specificity and use this loop in CD1d binding, this Vα24− TCR does not appear to rely heavily on its CDR3β loop for binding.

### Conformational Flexibility of Vα24− CDR3α Loops

**Figure 4:**
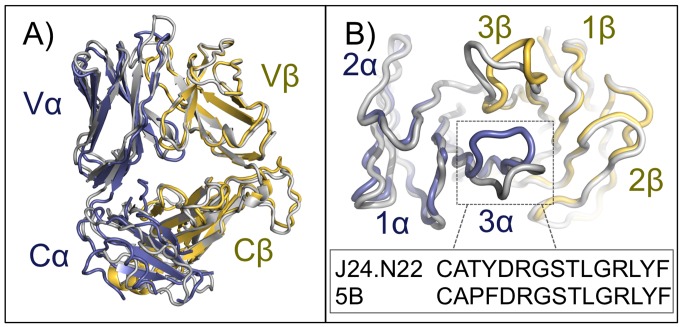
Superposition of unliganded and liganded Vα24− TCRs reveals CDR3 loop structural differences. (A) Superposition of the unliganded Vα24− (Vα3.1) TCR 5B (grey ribbon) [28] (PDB ID: 2CDG) and the Vα24− TCR of this study (α chain in light blue ribbon and β chain in yellow orange ribbon). (B) Close-up view of the α and β CDR loops of the unliganded and liganded Vα24− TCRs. The CDR loops are colored according to their TCR chain coloring in (A). The CDR3α loop sequences of the 5B and J24.N22 TCRs are shown at bottom.

The availability of a Vα24− TCR also expressing a Vα3.1 domain (named 5B) [28] in the unliganded state allows a direct comparison between the loop structures between the TCR examined here (bound to CD1d) and a Vα24−, Vα3.1+, TCR in its unbound state. Due to the use of different Jβ gene segments that results in global domain orientation shifts, the TCRs are not perfectly superimposable (Figure 4A) and there are two amino acid differences in the CDR3α sequences of these TCRs due to junctional diversity (Figure 4B). Alignment of the two Vα3.1 domains shows the CDR1 and CDR2 loops are essentially identical structurally (Figure 4B), yet examination of the CDR3α loops (Figure 4B) shows significant structural differences. While the unliganded structure of J24.N22 is not known, modeling of the 5B TCR onto our complex structure suggests a large shift in loop conformation would need to occur in the CDR3α loop for it to dock onto CD1d/αGalCer in a similar fashion. Because of the similarities between these TCRs in all other loops save the CDR3β, it is very likely that the 5B TCR would dock in a similar fashion as seen here. Thus in contrast to the Vα24+ NKT TCRs' recognition of CD1/αGalCer, where loop conformation was highly conserved in the liganded and unliganded state, we suggest that the CDR3α loop can be flexibile in Vα3.1+, Vα24− TCRs, similar to what was previously seen in the iNKT TCR recognition of CD1d/LPC [25].

### Residues Contributing to Vα24− TCR Binding of CD1d/αGalCer

**Figure 5:**
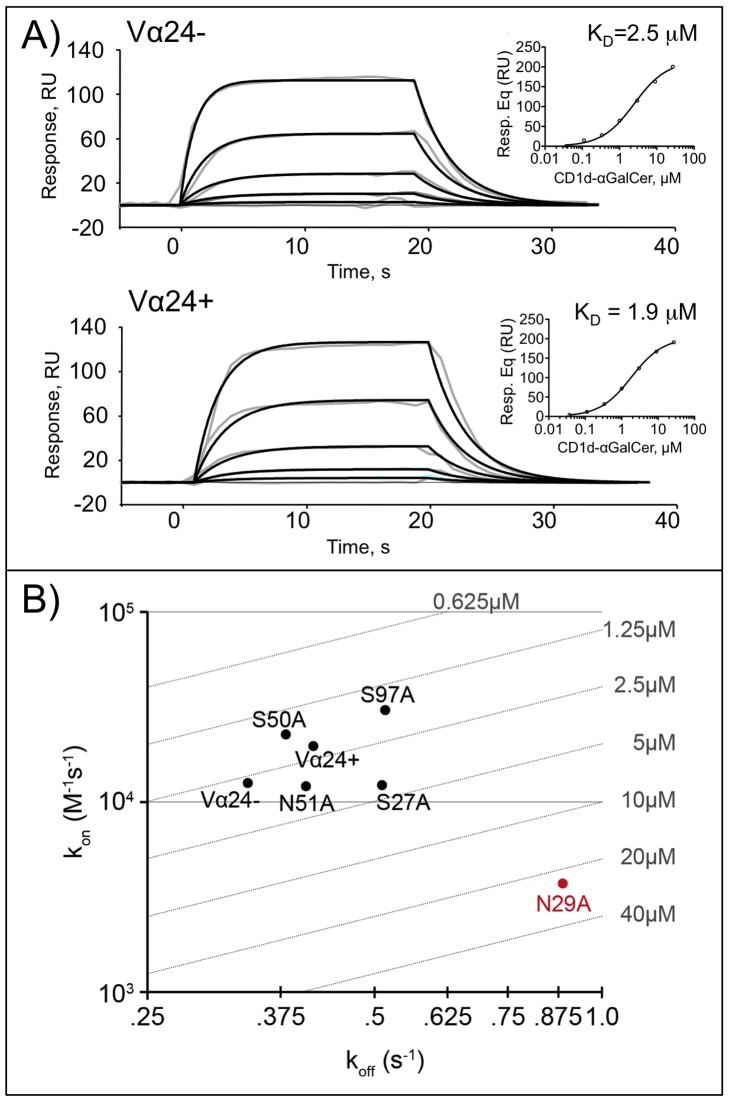
SPR analysis of the binding of CD1d-αGalCer to the Vα24− and Vα24+ NKT TCRs. (A) SPR binding curves of CD1d-αGalCer. Shown are the curves and fits used for kinetic analysis to surface-immobilized Vα24− (clone J24N.22) (top) and Vα24+ (clone J24L.17) (bottom) NKT TCRs. For the Vα24− TCR: k_on_ = 1.62×10^5^±0.11×10^5^ (Ms)^−1^, k_off_ = 0.342±0.007 s^−1^, and K_D_ = 2.1 µM; for Vα24+ TCR: k_on_ = 1.94×10^5^±0.16×10^5^ (Ms)^−1^, k_off_ = 0.414±0.010 s^−1^, and K_D_ = 2.1 µM. Grey traces represent experimental data and black lines fittings to a Langmuir 1∶1 kinetic model. Curves represent the following concentrations of analyte: 0, .037, .111, .333, 1, and 3 µM. In addition, equilibrium analysis was performed on these curves and those for 9 µM and 27 µM; the fits and calculated K_D_s for this analysis are shown as inserts. (B) Alanine scanning mutants of key residues in the Vα24− TCR interface were screened by SPR, and their k_on_ and k_off_ values are shown plotted on a k_on_ versus k_off_ plot with K_D_ isotherms shown along with the values for the wild-type Vα24− TCR and the Vα24+ iNKT TCR (J24.L17).

To evaluate the kinetics involved in binding of our Vα24− TCR with CD1d/αGalCer, we used surface plasmon resonance to measure the association (k_on_) and dissociation rates (k_off_) of this interaction and determine the dissociation constant (K_D_) (Figure 5A). We also used this to calculate K_D_ by equilibrium analysis (Figure 5A, insets). We included an iNKT (Vα24+) TCR in our kinetic measurements such that we could compare these values to a representative of the iNKT population. The affinity of the Vα24− TCR used in this study for CD1d/αGalCer (2.1 µM kinetic, 2.5 µM equilibrium) was similar to the affinity we measured for the iNKT TCR (2.1 µM kinetic, 1.9 µM equilibrium) as well as affinities from previous measurements with Vα24− TCRs (using Vα3.1 and Vα10.3 domains) [28]. Stronger affinities (0.5 µM) have been noted for other human iNKT TCRs [17].

We sought to further evaluate the residues contributing most to Vα24− TCR binding to CD1d/αGalCer. We chose key TCR residues identified as interacting with CD1d/αGalCer in our complex and evaluated their contribution to binding via alanine-scanning mutagenesis and SPR. We first evaluated the CDR1α loop residues Ser27 and Asn29, as these appeared to mediate the side-chain-specific contacts that differed most from the Vα24+ TCRs. While mutation of Ser27 to Ala (S27A) did not drastically change Vα24− TCR binding kinetics, mutating Asn29 to Ala (N29A) resulted in a significant disruption to binding with changes in both the association and dissociation rates and an increase in the K_D_ by an order of magnitude (Figure 5B). Thus the CDR1α loop provides a clear contribution to Vα24− TCR binding to CD1d/αGalCer. Previous mutational analysis of the CDR1α loop of a Vα24+ TCR [17] of Pro28 to Alanine disrupted binding, however this was assumed to be due to changes in the TCR architecture as conformational-specific antibodies failed to bind this mutant.

Mutation of the CDR2α side chains Ser50 and Asn51 had subtle effects on k_on_ and k_off_ (Figure 5B) yet did not appear to have a substantial effect on the overall affinity of CD1d/αGalCer binding, similar to what we observed with mutation of Ser97 in the CDR3β loop sequence. Because of the similarities in CDR3α loop contacts between Vα24− and Vα24+ TCRs, we included a mutation of Arg95 of the CDR3α as a positive control; this side chain has been shown to be central to iNKT TCR binding to CD1d/αGalCer [17]. We also observed that mutation of this side chain to Ala (R95A) abrogated binding of the Vα24− TCR and thus supports the importance of the CDR3α loop to Vα24− TCR docking.

## Discussion

Our complex structure of a Vα24− TCR with CD1d/αGalCer provides a model by which to understand how this diverse population of CD1d-restricted human T cells recognize antigen. These cells differ from iNKT cells in their specificity, effector function, and the markers expressed on their cell surface; these factors combined argue that these cells provide another arm of T-cell-mediated lipid recognition in humans. Here we provide a structural and biophysical foundation upon which to understand the molecular basis of differential reactivity observed at the cellular level in this NKT cell population.

Despite the divergent amino acid sequences encoded by the Vα3.1 domain for the CDR1α and CDR2α loops, the Vα24− TCR adopts a similar footprint to that of Vα24+ iNKT TCRs. This docking orientation is primarily dictated by the conserved docking of the CDR3α loop, containing the highly similar sequence encoded by the Jα18 segment of iNKT TCRs. The contacts mediated by the other loops, while not identical to those of iNKT TCRs, were very similar, suggesting that despite sequence differences in the Vα loops they could establish contacts with similar regions of the CD1d/αGalCer surface. The αGalCer headgroup position was almost identical to that observed in the iNKT complex structures [16],[19]. This docking mode, also shared with that of the murine Vα10 NKT TCR [11], is strikingly different from that of the recently resolved type II NKT TCR structures [9],[10], where the TCRs dock on an entirely different surface of CD1d (the A′ pocket) and use all six of the TCR's CDR loops in recognition (similar to what is observed in conventional αβ TCR recognition of MHC/peptide). These structures demonstrate that CD1d-restricted T cells can use at least two divergent ways to recognize their antigens [29].

Our complex structure provides a useful model to compare other Vα24− TCRs' structures, notably the structure of a highly related unliganded TCR called 5B [28]. If we assume the 5B TCR would dock similarly to the Vα24− TCR examined in our study, a significant conformational change would have to occur in 5B's CDR3α loop. This conformational flexibility was a feature we also observed in human iNKT TCR binding to CD1d/LPC [25]. In contrast to what was observed with the iNKT TCR complex structure with CD1d/αGalCer [16],[19], this suggests that not all CD1d-TCR interactions are “lock and key” and that changes to CDR3α loop conformation may contribute to differences in binding kinetics and thermodynamics. A similar phenomenon of loop movement was observed in the murine Vα10 NKT TCR upon binding [11].

The CDR3α loop footprint on CD1d/αGalCer is conserved in all the iNKT-TCR/CD1d structures noted to date as it is here. However, the number of contacts in this complex structure were less than that observed in the iNKT-TCR CD1d/αGalCer complex structure, yet the binding affinities measured for the Vα24+ and Vα24− TCRs in this study did not differ substantially (∼2 µM for both TCRs). The alanine-scanning mutagenesis revealed important contributions from the CDR1α loop (in particular, residue N29) in the Vα24− TCR binding that were not noted in Vα24+ TCR binding (mutation of the equivalent position, S30 in the Vα24+ TCR, showed little effect [17]). This shift of importance toward the CDR1α likely compensates for fewer CDR3α loop contacts and would explain the altered reactivity patterns of Vα24− TCRs for lipids that are recognized similarly by Vα24+ TCRs (such as αGlcCer and αGalCer, discussed more below). We cannot rule out that contributions from other loops, such as the CDR2α and CDR3β, contribute as well; while individual mutagenesis of these residues had small effects upon TCR binding, in combination they may have a cumulative effect in binding CD1d/lipid, evident only when they are mutated in concert.

Extensive studies in the mouse iNKT cell system have revealed how lipid ligands are structurally modified during recognition by the iNKT TCR. Even though extensive structural variability exists in the glycolipid headgroups, each carbohydrate structure adopts a similar orientation when bound by the TCR [20]–[24]. Therefore, contributions of the CDR1α in recognition of alternative lipids, both α- and β-linked glycolipids, could be an important factor in Vα24− T cell reactivity towards different lipids. Directly relevant to this point is the clear distinction between Vα24− T cells and Vα24+ iNKT cells in their differential reactivity to the α-linked glycolipids αGlcCer and αGalCer. Vα24+ iNKT cells respond well to both lipids, whereas Vα24− T cells do not respond to αGlcCer. The only difference present between these two lipids is the orientation of the 4′OH group on the sugar ring (glucose versus galactose). Our structural and biophysical data provide an explanation for this difference in reactivity. Asn29, a residue in the Vα24− CDR1α, establishes both VDW and hydrogen bonds with the 3′OH and 4′OH. Mutation of this residue to alanine results in an order of magnitude decrease in binding of the Vα24− TCR, presumably due to disruption of these contacts. Furthermore, the CDR2α loop residues Ser50 and Asn51 establish water-mediated hydrogen bonds with the 4′OH that may help to stabilize the interaction despite lacking clear energetic contributions (as assessed in our alanine-mutagenesis studies). We therefore propose that modification to the 4′OH between the galactose (αGalCer) and glucose (αGlcCer) structure is the primary molecular factor mediating the differences in reactivity of the Vα24− population of CD1d-restricted T cells. The alternative contacts with the carbohydrate headgroup in the iNKT TCR/CD1d/αGalCer structure may explain why iNKT cells can respond to both lipids; the main contacts with the 4′OH are mediated by Ser30, which when mutated to alanine only had a minimal effect on binding [17]. The greater number of contacts and BSA of the Vα24+ TCR CDR3α loop on CD1d/αGalCer may make these T cells relatively insensitive to variation in the glycolipid headgroup at other positions. The difference in 4′OH recognition may translate to alternative reactivity to other glycolipid and non-glycolipid lipid structures both in development of these T cells in the thymus and their effector functions in the periphery. Despite their shared use of Jα18 and Vβ11, the Vα24− T cells are differentiated from iNKT cells in their development and activation state; presumably altered TCR recognition of a selecting antigen during thymic development plays a role in these differences. Our structure provides a model by which to understand the molecular basis of this altered reactivity.

Our results, which focus much of the differences in reactivity to αGlcCer on the CDR1α loop and its interaction with the 4′OH, contrast with the murine Vα10 NKT cell preferred reactivity to αGlcCer [11], where preference in binding appears due to many factors. The highly convergent recognition of αGlcCer by these TCRs distributes the binding contacts over much of the CDR loop surfaces [11]. While mutagenesis data for these residues are not available, it is clear there are differences in the nature of the contacts between the Vα10 and iNKT TCRs with CD1d (VDW versus hydrogen bonds), that many new contacts are established with CD1d, and therefore modification to the sugar ring may have more of a distributed effect over the Vα10 NKT interaction than what we observe in our Vα24− TCR complex structure. Both structures, however, provide molecular models for the observed differences in lipid reactivity and demonstrate how divergent NKT TCR structures can convergently recognize similar CD1d/lipid antigen structures. The molecular basis of the differences in recognition we have described here are the first clues into understanding why Vα24− cells are developmentally and functionally distinct from the iNKT population.

## Materials and Methods

### Human Wild-Type CD1d− β_2_m Expression and Purification

The ectodomain region of human CD1d and human β_2_microglobulin (β_2_m) were co-expressed in insect cells and purified as described [25].

### Vα24^+^ and Vα24^−^ TCR Expression and Purification

The cDNAs corresponding to the α and β chains of the Vα24^+^ NKT TCR clone J24L.17 and the α and β chains of Vα24− TCR clone J24N.22 were separately cloned into different versions of the pAcGP67A vector each containing a 3C protease site followed by either acidic or basic zippers and a 6xHis tag. Both chains were co-expressed in Hi5 cells via baculovirus transduction. The heterodimeric TCRs was captured with Nickel NTA Agarose (Qiagen) and further purified by anion exchange and size-exclusion chromatography.

### Generation of Vα24− TCR Mutants

Mutants of the Vα24− TCR (S27A, N29A, S50A, N51A, R95A for the alpha chain, and S97A for the beta chain) were generated through overlapping PCR with specific primers containing the desired mutation. Mutant heterodimeric TCR was expressed in insect cells as described above.

### CD1d Loading with αGalCer

Purified human CD1d was used for loading with αGalCer at room temperature with a three molar excess of lipid for 16 h. The excess of lipid was then removed with a Superdex 200 (10/30) column (GE Healthcare).

### Surface Plasmon Resonance Measurements

A human CD1d construct bearing a 3C protease site + 6X-Histidine tag at the C-terminus was expressed in Hi5 cells and purified as described [25]. All interaction experiments were performed in a BIAcore 3000 Instrument (GE Healthcare). Three hundred RUs of wild-type Vα24− NKT TCR or a mutant version of it were captured in a flow channel of an Ni-NTA sensor chip (GE Healthcare) previously treated with NiCl_2_. Insect-cell-derived recombinant IgFc was used to block unbound sensor chip surface to minimize nonspecific binding events. Increasing concentrations (0, 0.037, 0.111, 0.333, 1, 3, 9, and 27 µM) of CD1d–αGalCer were injected at a flow rate of 30 µl/min in 10 mM Hepes pH 7.4, 150 mM NaCl, and 0.005% Tween-20. Both kinetic and equilibrium parameters were calculated off of these curves using BIAevaluation software 3.2RC1 (GE Healthcare) and GraphPad Prism.

### Ternary Complex Formation and Crystallization

Nickel agarose-purified Vα24− TCR was digested with 3C protease for 16 h at 4°C to remove the zippers and His tags and purified by anion exchange chromatography in a MonoQ column (GE Healthcare). Endoglycosidase F3 (EndoF3) was used next at a 1∶10 enzyme-to-protein ratio for 2 h at 37°C in order to minimize the sugar content present in the protein. The digested protein was purified by a new round of anion exchange followed by size-exclusion chromatography. Both αGalCer-loaded CD1d and EndoF3-treated Vα24− TCR protein samples were mixed in HBS at 1∶1 molar ratio and concentrated in Nanosep Centrifugal Devices (Pall Life Sciences) to 10 mg/ml. Initial hits were found in 0.1 M sodium acetate, 20% PEG 4000, and were optimized to birefringent crystals that grew in 0.1 M sodium acetate pH 5.0, 17% PEG 4000, and 0.1 M ammonium acetate.

### Crystallographic Data Collection, Structure Determination, and Refinement

Crystals were cryo-cooled in mother liquor supplemented with 20% glycerol prior to data collection. All data sets were collected on a MarMosaic 300 CCD at the LS-CAT Beamline 21-ID-G at the Advanced Photon Source (APS) at Argonne National Laboratory and processed with HKL2000 [30].

The structure of the ternary complex was solved by molecular replacement with the program Phaser [31] using the human CD1d–β2m (Protein Data Bank (PDB) accession number 1ZT4) and an iNKT Vα24+ TCR (2EYS) as search models. Refinement with Phenix software suite [32] was initiated through rigid body and followed with XYZ coordinates and individual B-factor refinement. These first steps of refinement yielded clear unbiased and continuous density for αGalCer. Next, extensive cycles of manual building in Coot [33] and refinement were carried out and ligands such as αGalCer or covalently bound sugars were introduced guided by Fo−Fc positive electron density. Ligands structures and chemical parameters were defined with C.C.P.4.'s Sketcher [34] and included in subsequent refinement and manual building steps. Translation/libration/screw (TLS) partitions were calculated and incorporated at later refinement stages. All the refinement procedures were performed taking a random 5% of reflections and excluding them for statistical validation purposes (Rfree).

### Structure Analysis

Intermolecular contacts and distances were calculated using the program Contacts from the CCP4 software package [34], interface surface areas were calculated using the PISA server (http://www.ebi.ac.uk/msd-srv/prot_int/pistart.html), and all structural figures were generated using the program Pymol (Schrödinger, LLC).

### Accession Numbers

Coordinates and structure factors for the J24.N22 Vα24− TCR/CD1d/αGalCer complex have been deposited in the Protein Data Bank under the accession code 4EN3.

## Abbreviations

CD: cluster of differentiation
CDR: complementarity determining region
IFN: interferon
IL: interleukin
MHC: Major histocompatibility complex
PDB: Protein Data Bank
SPR: surface plasmon resonance
V: variable
